# Brain atrophy and clinical characteristics predicting SDMT performance in multiple sclerosis: A 10-year follow-up study

**DOI:** 10.1177/2055217321992394

**Published:** 2021-02-08

**Authors:** Cecilie Jacobsen, Robert Zivadinov, Kjell-Morten Myhr, Turi O Dalaker, Ingvild Dalen, Ralph HB Benedict, Niels Bergsland, Elisabeth Farbu

**Affiliations:** Department of Neurology, Stavanger University Hospital, Stavanger, Norway; Department of Clinical Medicine, University of Bergen, Bergen, Norway; Buffalo Neuroimaging Analysis Center, Department of Neurology, Jacobs School of Medicine and Biomedical Sciences, University at Buffalo, State University of New York, Buffalo, NY, USA; Department of Clinical Medicine, University of Bergen, Bergen, Norway; Neuro-SysMed, Department of Neurology, Haukeland University Hospital, Bergen, Norway; Stavanger Medical Imaging Laboratory (SMIL), Department of Radiology, Stavanger University Hospital, Stavanger, Norway; Section of Biostatistics, Department of Research, Stavanger University Hospital, Stavanger, Norway; Buffalo Neuroimaging Analysis Center, Department of Neurology, Jacobs School of Medicine and Biomedical Sciences, University at Buffalo, State University of New York, Buffalo, NY, USA; Buffalo Neuroimaging Analysis Center, Department of Neurology, Jacobs School of Medicine and Biomedical Sciences, University at Buffalo, State University of New York, Buffalo, NY, USA; IRCCS, Fondazione Don Carlo Gnocchi ONLUS, Milan, Italy; Department of Neurology, Stavanger University Hospital, Stavanger, Norway; Department of Clinical Medicine, University of Bergen, Bergen, Norway

**Keywords:** Atrophy, biomarkers, cognition, longitudinal, MRI, multiple sclerosis

## Abstract

**Objectives:**

To identify Magnetic Resonance Imaging (MRI), clinical and demographic biomarkers predictive of worsening information processing speed (IPS) as measured by Symbol Digit Modalities Test (SDMT).

**Methods:**

Demographic, clinical data and 1.5 T MRI scans were collected in 76 patients at time of inclusion, and after 5 and 10 years. Global and tissue-specific volumes were calculated at each time point. For the primary outcome of analysis, SDMT was used.

**Results:**

Worsening SDMT at 5-year follow-up was predicted by baseline age, Expanded Disability Status Scale (EDSS), SDMT, whole brain volume (WBV) and T2 lesion volume (LV), explaining 30.2% of the variance of SDMT. At 10-year follow-up, age, EDSS, grey matter volume (GMV) and T1 LV explained 39.4% of the variance of SDMT change.

**Conclusion:**

This longitudinal study shows that baseline MRI-markers, demographic and clinical data can help predict worsening IPS. Identification of patients at risk of IPS decline is of importance as follow-up, treatment and rehabilitation can be optimized.

## Introduction

Multiple sclerosis (MS) is a chronic inflammatory and neurodegenerative disease, characterized by multifocal areas of demyelination and atrophy of the central nervous system (CNS).^1^ These pathological changes are seen both in the white matter (WM), and the grey matter (GM) of the CNS.^2^

Cognitive impairment (CI) in MS has been increasingly investigated over the past decades, and is acknowledged as a major symptom present in a large proportion of patients, with a prevalence in the range of 40–70% in cross-sectional MS-populations.^3^ CI affects patients in all stages and subtypes of the disease, even from the prodromal phase^4^ and the early stage of clinically isolated syndrome^5^ to those living with the disease for several decades.^6^ The cognitive domains most commonly affected are information processing speed and episodic memory,^3^ yet impairment of any cognitive domain could be present.^7^

Previous studies have shown that MS patients with CI have a lower chance of being employed, are less likely to engage in social activities and found household tasks more difficult. They are also more likely to suffer from psychiatric illness and have lower quality of life scores.^8^ MS CI is associated with physical disability, as measured by the Expanded Disability Status Scale (EDSS),^9^ and predicts later EDSS worsening.^10^

MRI biomarkers associated with CI have been extensively investigated over the past decades.^11^ The search for reliable radiological biomarkers has yielded numerous studies shedding light on the association between lesion volume (LV), atrophy and CI.^12–14^ Cerebral atrophy, T2 LV and cortical lesions have been identified as possible culprits, all of which result in an increased risk of CI.^15–17^

Neuropsychological test batteries for investigating CI in MS are numerous. However, the Symbol Digit Modalities Test (SDMT) is one of the most commonly used tests for assessing information processing speed (IPS), an essential cognitive function. When IPS is affected, downstream processes may be influenced, such as memory, executive functions, learning and word retrieval.^18^ SDMT is increasingly attractive for use in the clinical setting as well as research due to its fast and easy administration, excellent test-retest reliability, good validity and high sensitivity to CI in MS.^18^ A clinical meaningful change of SDMT has been proposed in several studies, with a raw score change of 4 points or a 10% change to be suggestive of cognitive decline.^18,19^

Our aim is to reveal clinical, demographic and MRI measures predictive of worsening IPS as measured by change in SDMT. We also aimed to explore parameters predictive of a clinically meaningful change of SDMT. Our hypothesis was that grey matter atrophy would be predicitive of worsening IPS.

## Methods

### Patients

In the years of 1998–2000 patients diagnosed with multiple sclerosis at the Haukeland University Hospital (HUS) and Stavanger University Hospital (SUS) in the south-western parts of Norway were given the opportunity to enter into the study. Patients were included at time of diagnosis, and re-examined after 5- and 10-years. The current diagnostic criteria at the time of enrolment, the criteria of Poser, were used to establish the diagnosis of MS.^20^

A total of 108 patients qualified for inclusion. From those, three patients had moved out of the area, one was deceased and 11 declined participation, leaving 93 patients. Neurological examination, MRI of the brain and the required tests were performed in 76 of these patients, and they were subsequently included in the present study. The cohort comprised of patients with all MS-subtypes.

After 5- and 10 years the patients were re-examined, including MRI of the brain, clinical and cognitive assessment.

Physical disability was assessed using the EDSS at each visit.

Level of education at baseline was registered. The patients were classified as having low (12 years or less; primary school/junior high), or high (more than 12 years; college/university) level of education.

The regional committee for medical and health research of western Norway, the Norwegian Centre for Research Data and the Norwegian Data Protection Authority approved the study. All patients signed an informed written consent in accordance with the Helsinki convention.

### MRI acquisition and analysis

The MRI scans were completed at two different scanners, one located at HUS (Siemens Symphony), and one at the SUS (Phillips Medical systems, Intera). The same standardized study protocol was used at each time-point. The scanner strength was 1.5 T and the protocol used consisted of a dual spin echo (SE) proton density (PD)/T2–weighted image (WI), a three-dimensional (3 D) T1-WI and a SE-T1-WI. The voxel size for (SE) PD/T2-WI was 0.9 × 0.9 × 5.0 mm^3^, for 3 D T1-WI 0.9 × 0.9 × 1.4 mm^3^, and for SE T1 0.9 × 0.9 ×5.0 mm^3^ on the Siemens scanner. On the Philips scanner the voxel size for (SE) PD/T2-WI was 0.89 × 0.89 × 5.0 mm^3^, for 3 D T1-WI 0.89 × 0.89 ×1.2 mm^3^, and for SE T1 0.89 × 0.89 × 5.0 mm^3^.

The protocol is described in detail elsewhere.^21^

In order to calculate global and tissue-specific atrophy measures and lesion volumes, the MRI scans were subsequently analyzed. Using the FMRIB’s FLIRT (Functional MRI of the Brain’s Linear Image Registration Tool), all baseline and follow-up scans for each subject were co-registered to its baseline T1 SE image. Next, using the co-registered images, T1 and T2 lesion volumes (LVs) were calculated using a reliable, semi-automated edge detection contouring/thresholding technique previously described.^22^ Prior to performing further analysis on the 3 D-T1 scans, the lesion-filling tool from FSL was applied to minimize the impact of WM lesions on tissue segmentations.^23^ Normalised measures for whole brain volume (WBV), GM, WM, cortical volume (CV) and lateral ventricular volume (LVV) were measured using SIENAX (V2.6) as previously described.^24,25^

From the inpainted 3 D-T1 images, absolute volumes of the subcortical deep grey matter (SDGM) structures were calculated using the FMRIB’s Integrated Registration and Segmentation Tool (FIRST V1.2), a model-based segmentation and registration tool.^26^ Normalised SDGM volumes were estimated by multiplying the estimated volumes from FIRST by the volumetric scaling factor from SIENAX.^24^

This process is described in detail elsewhere.^21^

### Cognitive evaluation

In order to define CI and cognitively preserved (CP) at each time point, the patients underwent neuropsychological testing as follows:

- Paced Auditory Serial Addition Test (PASAT) assessing working memory,
- Selective Reminding Test (SRT) measuring working memory and learning, including sub-scores of long time storage (LTS) and delayed recall (DR)
- Symbol Digits Modalities Test (SDMT) measuring cognitive processing speed

We defined CI as scoring below 1.5 standard deviations (SD) compared to a healthy control group on two or more tests. A control group consisting of 40 persons was recruited from the staff at SUS. When comparing to the baseline patient group, the control group were similar in age (42.4; SD 12.6; p = 0.77) and sex (26 female, 56%; p = 0.53) More persons in the control group had higher education compared to the patient group (68% vs 34%; p = 0.001). .

SDMT has become highly recommended as the primary cognitive test in MS, thus we chose SDMT score as the cognitive outcome measure in the regression analyses.^18^

### Statistical analysis

Descriptive statistics were performed using SPSS V.26 (Armonk, NY, USA: IBM Corp.). Results are presented as means and SD, medians and interquartile ranges (IQRs) or as counts and percentages for continuous symmetric, continuous non-symmetric and categorical data, respectively.

Baseline predictors of change in SDMT during follow-up were assessed in linear regression models. Results from univariable and multivariable models are presented as unstandardized β values with 95% confidence intervals (CI) and p-values from Wald tests. As a measure of goodness of fit or predictive power we present R^2^, and the change in R^2^ (ΔR^2^) as a measure of the improvement of the model when including a predictor. Due to limited sample sizes, we performed the multivariable modelling stepwise, i.e. by first finding an optimal set of clinical and demographic variables with high predictive power (using manual backwards elimination and subsequent forward inclusion; model fit evaluated by the adjusted R^2^), and this model acted as a base model for which we evaluated added predictive value from each MRI variable. Finally, the MRI variables that were most predictive were tried in combination, and a “best” model decided upon by adjusted R^2^ (always keeping the demographic and clinical variables in the model). Similarly, predictors of clinically meaningful change in SDMT (i.e. >4 points reduction) were evaluated in logistic regression models, from which we report odds ratios (OR) with 95% CI, p-values from Wald tests, and with Nagelkerke pseudo R^2^ and the C-index as measures of predictive performance.

All regression analyses were performed in Stata v. 16.1 with functions regress, logit, roctab and fitstat. P-values <0.05 were considered statistically significant.

## Results

### Demographic, clinical and MRI data at baseline, 5- and 10- year follow up

**Figure 1.**
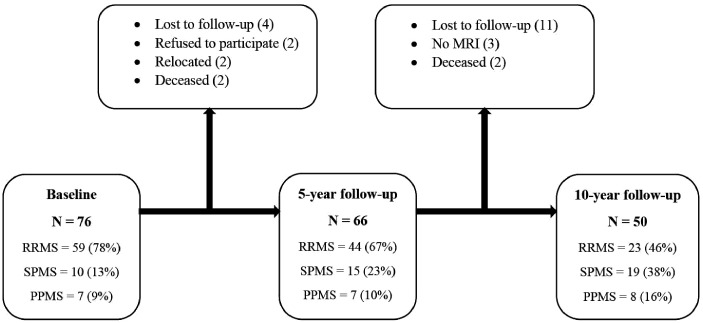
Flow chart of patient inclusion at baseline, 5-year and 10-year follow-up.

Baseline demographic, clinical and MRI characteristics categorized by cognitive status, of the patient groups at baseline, 5- and 10-year follow-up are shown in Table 1. At baseline 37 of 76 (49%) of the patients were classified as cognitively impaired. The number at 5-year follow up was 28 of 60 (47%) and at the 10-year follow-up 14 of 38 (37%). After 5 years of follow-up, 66 patients were re-examined while 50 patients remained at the 10-year follow up (Figure 1). Of the patients classified as CI at baseline 67.5% dropped out during the course of the follow-up, comparably 51.3% of the patients classified as (CP) dropped out during the follow-up.

**Table 1.** Baseline demographics, clinical characteristics and brain volumes split by cognitive status at baseline, 5- and 10-year follow-up.

	Baseline (n = 76)				5-year follow-up (n = 60)			10-year follow-up (n = 38)		
	All patients	Cognitively Impaired	Cognitively Preserved	p	Cognitively Impaired	Cognitively Preserved	p	Cognitively Impaired	Cognitively Preserved	p
No. of patients (%)	76	37 (48.7)	39 (51.3)		28 (46.7)	32 (53.3)		14 (36.8))	24 (63.2)	
Age, mean (SD)	41.8 (9.7)	44.1 (10.2)	39.7 (8.9)	**0.046**	43.2 (8.6)	38.3 (9.3)	**0.039**	40.3 (7.7)	41.3 (9.5)	0.74
Female, n (%)	52 (68.4)	22 (59.5)	30 (76.9)	0.082	18 (64.3)	22 (68.8)	0.71	9 (64.3)	18 (75.0)	0.48
EDSS, median (IQR)	3.5 (2.57–4.0)	3.5 (3.0–4.3)	3.5 (1.5–4.0)	0.085	3.5 (3.1–4.5)	2.5 (1.5–3.5)	**<0.001**	3.5 (2.5–3.8)	3.25 (1.5–3.9)	0.75
MS subtype, n (%)				**0.026**			0.45			0.19
RRMS	59 (77.6)	28 (75.7)	31 (79.5)		21 (75.0)	28 (87.5)		14 (100)	19 (79.2)	
SPMS	10 (13.2)	8 (21.6)	2 (5.1)		3 (10.7)	2 (6.3)		0	1 (4.2)	
PPMS	7 (9.2)	1 (2.7)	6 (15.4)		4 (14.3)	2 (6.3)		0	4 (16.7)	
Disease duration, median (IQR)	60 (39–141)	84 (48–192)	48 (36–72)	**0.025**	72 (51–141)	48 (36–60)	**0.004**	66 (48–228)	48 (36–96)	0.14
DMT use, n (%)	11 (14.5)	6 (16.2)	5 (12.8)	0.46	13 (46.4)	12 (37.5)	0.34	10 (71.43)	16 (66.7)	0.44
High education, n (%)	26 (32.1)	7 (18.9)	17 (43.6)	**0.021**	5 (17.9)	12 (37.5)	0.080	4 (28.6)	9 (37.5)	0.58
SDMT, mean (SD)	42.1 (12.8)	33.2 (8.5)	50.8 (9.9)	**<0.001**	35.9 (9.0)	51.7 (9.9)	**<0.001**	34.7 (10.7)	49.7 (8.5)	**<0.001**
Cortical volume, mean (SD)	574.4 (46.2)	553.6 (41.2)	594.1 (42.3)	**<0.001**	557.5 (40.2)	599.7 (41.5)	**<0.001**	569.3 (49.2)	596.5 (40.5)	0.093
SDGM volume, mean (SD)	44.7 (5.6)	43.3 (5.8)	46.1 (5.2)	**0.034**	43.1 (4.7)	47.7 (5.0) [	**0.001**	43.5 (5.0)	46.3 (5.2)	0.12
T2 LV, median (IQR)	10.1 (2.7–20.9)	15.9 (6.3–31.4)	5.0 (1.6–13.8)	**0.003**	16.3 (7.4–28.9)	3.2 (1.4–11.0)	**<0.001**	21.2 (7.5–9.8)	3.8 (0.9–12.8)	**<0.001**
T1 LV, median (IQR)	3.0 (0.6–7.4)	4.2 (1.9–13.7)	1.2 (0.3–3.8)	**<0.001**	4.1 (2.2–10.6)	1.2 (0.2–3.3)	**<0.001**	5.4 (2.1–11.5)	0.8 (0.1–2.9)	**<0.001**

Note: The differences between the cognitively preserved and cognitively impaired were calculated using Chi square test, Student t test and Mann-Whitney rank sum test, as appropriate.

High education = College/university, Disease duration given in months. Brain volumes are presented in millilitres as mean, (SD).

EDSS: expanded disability status scale; MS: multiple sclerosis; RR: relapsing-remitting; SP: secondary progressive; PP: primary progressive; DMT: disease modifying therapy; SDMT: symbol digit modalities test; SDGM: subcortical deep grey matter; LV: lesion volume.

DMT use, n (%); numbers represent use at each time-point. Bold values denotes statistical significance at the p < 0.05 level.

Clinical and demographic characteristics of the patient group classified as CI at baseline included older age, higher EDSS score, longer disease duration, lower education and lower SDMT score at baseline compared with the CP group. At 5-year follow-up the CI patient group were significantly older, had a longer disease duration and a higher baseline EDSS.

At baseline and 5-year follow up, the CI group had significantly higher T1 and T2 LV, and lower cortical and SDGM volumes.

When comparing the two patient groups followed at SUS and HUS, we found no significant differences in clinical, demographic or MRI-parameters between the groups. For the patients included at HUS, although not significantly, a longer baseline disease duration of median 60 months (IQR 48–180), compared to SUS patients with a disease duration of median 48 months (IQR 36–84), (p = 0.07). Use of disease modifying therapy (DMT) at 5-year follow-up were median 2.3 months (IQR 0 – 62) at HUS, and 0 (IQR 0–9.9, p = 0.1) at SUS. At 5-year follow-up 21 of 40 (52.5%) of HUS patients, and 13 of 31 (41.9%) of SUS patients received DMTs. There was a greater dropout of patients at SUS 19 of 32 (59%) vs HUS 19 of 44 (43%).

### Baseline MRI and clinical variables predicting change of SDMT

Univariable linear regression analysis showed significant associations between baseline WBV, GMV, WMV, CV and T2 LV and change in SDMT during 5-year follow-up from baseline, where lower brain volume and higher LV predicted reduction of SDMT. Of the clinical and demographic variables, we found the combination of age, baseline EDSS and SDMT to explain 12.6% of the variance of change in SDMT. Being older, and having a higher EDSS and SDMT score was associated with a greater negative SDMT change. When adding individual MRI-parameters, WBV, WMV, LVV, T2 and T1 LV contributed significantly to this model. When trying combinations of WBV, LVV, T2 and T1 LV together with the selected clinical and demographical variables, the best model included baseline WBV and T2 LV and explained 30.2% of the variation in SDMT change. Being older and having a lower EDSS score was associated with a greater negative SDMT change (Table 2).

**Table 2.** Prediction of change in SDMT during 5 years from baseline (n = 66 all analyses).

	Univariable			Multivariable			
Baseline predictor	β (95% CI)	p	R^2^(%)	β (95% CI)	p	R^2^(%)	ΔR^2^(%)
				*Best model clin/dem*			
Age	–2.2 (–4.8, 0.4)	0.094	4.3	–1.8 (–4.5, 0.9)	0.20		
Sex (female)	1.6 (–3.8, 6.9)	0.56	0.5				
Center (SUS)	–6.1 (–11.0, –1.3)	0.014	9.0	–6.1 (–10.8, –1.4)	0.011		
Education (high)	2.4 (–3.0, 7.8)	0.38	1.2			21.6	
Log disease duration	–1.3 (–4.8, 2.1)	0.43	1.0				
MS type progressive	–3.8 (–10.1, 2.5)	0.23	2.2				
EDSS	–1.8 (–3.7, 0.1)	0.057	5.6	–1.8 (–3.9, 0.3)	0.084		
SDMT	–0.11 (–0.31, 0.08)	0.25	2.0	–0.20 (–0.40, –0.01)	0.043		
				*When added to best model clin/dem*			
Whole brain	0.043 (0.015, 0.071)	**0.003**	12.7	0.042 (0.009, 0.076)	**0.015**	29.0	7.4
Grey matter	0.05 (0.01, 0.10)	**0.022**	8.0	0.05 (–0.00, 0.11)	0.053	26.4	4.8
White matter	0.08 (0.02, 0.13)	**0.007**	11.0	0.06 (0.00, 0.13)	**0.036**	27.1	5.5
Ventricular	–0.05 (–0.17, 0.06)	0.36	1.3	–0.17 (–0.31, –0.03)	**0.015**	29.0	7.4
Cortical	0.07 (0.02, 0.13)	**0.007**	10.9	0.06 (–0.00, 0.13)	0.065	27.7	6.1
T2 LV	–0.22 (–0.42, –0.02)	**0.028**	7.3	–0.35 (–0.55, –0.16)	**0.001**	35.7	14.1
T1 LV	–0.37 (–0.75, 0.02)	0.060	5.4	–0.51 (–0.91, –0.12)	**0.012**	29.5	7.9
Subcortical	0.29 (–0.17, 0.75)	0.21	2.5	0.30 (–0.19, 0.80)	0.22	23.5	1.9
Caudate	0.5 (–2.3, 3.2)	0.73	0.2	–0.2 (–3.0, 2.7)	0.91	21.6	0.0
Putamen	1.0 (–1.0, 2.9)	0.33	1.5	1.1 (–1.0, 3.2)	0.29	23.1	1.5
Thalamus	1.2 (–0.2, 2.5)	0.094	4.3	1.2 (–0.3, 2.7)	0.11	24.9	3.3
Pallidus	–0.8 (–6.4, 4.8)	0.79	0.1	0.9 (–4.9, 6.6)	0.77	21.7	0.1
Hippocampus	1.4 (–0.9, 3.6)	0.23	2.2	1.5 (–0.8, 3.8)	0.20	23.8	2.2
Amygdala	4.7 (–0.7, 10.1)	0.088	4.5	3.3 (–2.2, 8.9)	0.24	23.4	1.8

MS: multiple sclerosis (progressive=primary and secondary progressive combined); EDSS: expanded disability status scale; SDMT: symbol digit modalities test; LV: lesion volume; CI: confidence interval.

Note: Effect estimates from linear regression analysis with change in SDMT as outcome (given as SDMT at 5 years minus SDMT at baseline), and baseline variables as predictors. A positive β value means that a higher value of the predictor is associated with a higher change value, i.e. a slower decline of cognitive processing speed. A negative β means that a higher value of the predictor is associated with a greater negative change of SDMT, i.e. a faster decline of cognitive processing speed. R^2^ estimates the predictive power of each model, and ΔR^2^ the contribution to the model from each predictor.

Bold values denotes statistical significance at the p < 0.05 level.

Similar analysis was done during the 10-year follow-up. The univariable linear regression analysis showed a lower WBV, GMV, CV, SDGM volume, and higher T1 and T2 LV to be significantly predictive of SDMT reduction during 10-year follow-up. Age, center and baseline EDSS together explained 6.2% of the variance in SDMT change. Out of GMV, CV, T2 and T1 LV, the combination of T1 LV and GMV explained the most of the variance together with the clinical/demographical variables, which explained 39.4% of the SDMT change variance (Table 3).

**Table 3.** Prediction of change in SDMT during 10 years from baseline (n = 50 all analyses).

	Univariable			Multivariable			
Baseline predictor	β (95% CI)	p	R^2^(%)	β (95% CI)	p	R^2^(%)	ΔR^2^(%)
				*Best model clin/dem*			
Age	–1.8 (–4.9, 1.4)	0.27	2.6	–3.0 (–6.4, 0.4)	0.083		
Sex (female)	0.6 (–6.0, 7.2)	0.86	0.1				
Center (SUS)	–7.8 (–13.8, –1.9)	0.011	12.7	–7.9 (–13.7, –2.0)	0.009	18.9	
Education (high)	1.7 (–4.5, 7.8)	0.59	0.6				
Log disease duration	–1.6 (–5.6, 2.3)	0.40	1.5				
MS type progressive	0.3 (–7.6, 8.3)	0.93	0.0				
EDSS	0.6 (–1.6, 2.8)	0.60	0.6	1.7 (–0.7, 4.0)	0.16		
SDMT	–0.07 (–0.31, 0.17)	0.56	0.7				
				*When added to best model clin/dem*			
Whole brain	0.036 (0.002, 0.070)	**0.036**	8.8	0.035 (–0.001, 0.071)	0.057	25.2	6.3
Grey matter	0.06 (0.01, 0.11)	**0.018**	11.1	0.08 (0.02, 0.13)	**0.007**	31.2	12.3
White matter	0.03 (–0.04, 0.10)	0.38	1.6	0.01 (–0.06, 0.08)	0.75	19.1	0.2
Ventricular	–0.04 (–0.17, 0.10)	0.58	0.6	–0.10 (–0.24, 0.04)	0.17	22.3	3.4
Cortical	0.08 (0.02, 0.13)	**0.011**	12.7	0.08 (0.02, 0.15)	**0.014**	29.2	10.3
T2 LV	–0.35 (–0.55, –0.15)	**0.001**	19.9	–0.42 (–0.60, –0.24)	**<0.001**	45.3	26.4
T1 LV	–1.0 (–1.5, –0.5)	**<0.001**	24.6	–1.1 (–1.5, –0.7)	**<0.001**	47.5	28.6
Subcortical	0.53 (0.01, 1.05)	**0.045**	8.1	0.57 (0.07, 1.07)	**0.026**	27.5	8.6
Caudate	2.0 (–1.2, 5.1)	0.22	3.1	2.1 (–1.0, 5.2)	0.19	22.0	3.1
Putamen	2.0 (–0.3, 4.3)	0.083	6.1	2.5 (0.4, 4.7)	**0.023**	27.8	8.9
Thalamus	1.5 (–0.0, 3.1)	0.054	7.5	1.5 (–0.0, 3.0)	0.050	25.6	6.7
Pallidus	3.0 (–3.7, 9.6)	0.38	1.6	5.0 (–1.2, 11.3)	0.11	23.4	4.5
Hippocampus	2.6 (0.1, 5.2)	**0.042**	8.3	2.6 (0.2, 5.0)	**0.033**	26.8	7.9
Amygdala	5.0 (–1.5, 11.6)	0.13	4.7	4.1 (–2.1, 10.4)	0.19	22.0	3.1

MS: multiple sclerosis (progressive=primary and secondary progressive combined); EDSS: expanded disability status scale; SDMT: symbol digit modalities test; LV: lesion volume; CI: confidence interval.

Note: Effect estimates from linear regression analysis with change in SDMT as outcome (given as SDMT at 10 years minus SDMT at baseline), and baseline variables as predictors. A positive β value means that a higher value of the predictor is associated with a higher change value, i.e. a slower decline of cognitive processing speed. A negative β means that a higher value of the predictor is associated with a greater negative change of SDMT, i.e. a faster decline of cognitive processing speed. R^2^ estimates the predictive power of each model, and ΔR^2^ the contribution to the model from each predictor.

Bold values denotes statistical significance at the p < 0.05 level.

We explored the effects of DMT, by adding DMT use at each timepoint dichotomized to active- and highly-active treatment. When adding DMT to the model, SDGM volumes were no longer significantly predictive of SDMT change at 10-year follow-up. No changes to the 5-year results was seen.

### Baseline MRI and clinical variables as predictors of clinically meaningful change of SDMT

Baseline EDSS, WBV, GMV, WMV, CV, T1 and T2 LV were significantly predictive of a clinically meaningful SDMT change of 4 points during 5-year follow-up in univariable logistic regression analysis. T2 LV contributed the most to the prediction of clinically meaningful SDMT change of more than 4 points, with an increase in Nagelkerke R^2^ of 6.6 percentage points and in c-index of 0.03 when included in the model with center, disease duration and EDSS at baseline (Supplementary Table 1).

Only univariable logistic regression analysis could be performed for clinically meaningful SDMT loss during 10-year follow-up, due to few cases. None of the clinical or demographic variables were predictive of clinically meaningful SDMT loss, and of the MRI-parameters T1 and T2 LV were the only statistically significant predictors of clinically meaningful SDMT loss (Supplementary Table 2).

## Discussion

This prospective, longitudinal study of a cohort of MS patients identified clinical and MRI-markers predicting worsening IPS, as measured by SDMT.

Nearly 50% of the patient group where classified as CI at baseline. This finding is in line with current knowledge, describing a prevalence of CI in MS ranging from 40% – 70%, depending on the group of patients studied, and test-strategies.^11^

As our patient group had a relatively long disease duration at baseline of close to 9 years, we suspect that effects due to brain atrophy as they relate to cognitive difficulties may have already ensued. Supporting this theory, is the fact that there was not an increase in the rate of CI over the 10-year follow up. Part of the reason for this, however, is probably due to drop-outs. Of the baseline CI patients 67.5% dropped out in the course of the follow-up, comparably 51.3% of the CP group dropped out (Figure 1).

One of the main findings of this study is that the constellation of age, EDSS, SDMT, WBV and T2 LV explained 30.2% of the variance in change of SDMT 5 years after diagnosis. Age, baseline EDSS, GMV and T1 LV explained 39.4% of the variance in 10-year SDMT change.

A great body of research is available, describing MRI-parameters associated with CI. WM lesions are found to be associated with CI in numerous studies, and specifically disruption of strategic WM tracts can cause CI among other clinical symptoms.^27^ However, damage to normal appearing WM and GM have shown stronger correlations to CI.^28^ Unfortunately though, we were unable to measure such damage with the imaging protocol utilized in this study.

Prediction of CI has been explored in a few studies showing lesion load, WB atrophy, diffuse brain damage and central atrophy.^17,29^ A recent study including 234 patients found cortical volume loss as the main driver, along with decreased anterior thalamic radiation integrity, to be the most significant predictors of cognitive decline^30^ Our findings support these studies, in showing both LVs and atrophy of WBV and GMV leading to worsening IPS.

Clinical and demographic determinants of CI was in a large study of 303 MS patients found to include disease duration, EDSS and vocabulary.^31^ Another recent study found age to be the only significant baseline predictor.^30^ We found similar results, and even if there was a significant difference between CI and CP patients regarding level of education, interestingly no predictive value of education at baseline was seen. This was in line with Eijlers et al,^30^ and may indicate that the protective effect of education, suggested to contribute to cognitive reserve, was already exhausted at baseline, as the patients had a quite long disease duration.

When investigating clinically meaningful SDMT loss of 4 points, EDSS, WBV, GMV, WMV and CV were significant, independent baseline predictors. The combination of disease duration, EDSS and T2 LV were the clinical parameters best predictive of SDMT loss of 4 points at 5-year follow-up. At the 10-year follow-up, only T1 and T2 lesion volumes were significantly predictive of SDMT loss of 4 points. Only 13 patients had a 4 point decline in SDMT, hence type II error could be the reason why none of the atrophy measures were statistically significantly associated.

A strength of this paper, is the fact that the patient group consist of an unselected cohort of MS patients followed for 10 years from time of diagnosis. The patients were mainly untreated for the first part of the follow-up, providing insight in the occurrence of brain atrophy and CI in the absence of newer, potent treatment options.

Some limitations need mentioning. We had a relatively small sample size, and a noticeable drop-out of (about 35 pts) over the 10-year follow up. The results of the 10-year follow-up group needs to be interpreted with care as the patient group is small.

Decrease in SDMT score was used as the primary outcome. However, some patients had improved SDMT score over the follow-up. At 5-year follow-up 26 of the 66 patients had an improved SDMT score, the total number of patients having an improved SDMT score at 10-year follow-up was 28 out of 50.

Scans were obtained using 1.5 T MRI systems, volumetric segmentations would have been more precise and reliable using a 3 T MRI. Performing MRI scans on two different scanners is a possible source of error, however, effects of different scanners on longitudinal volume changes are considered to be minor.^32^ It is essential to emphasize that this paper, for the main part, specifically investigated IPS decline, not CI at a broader level. Cognitive reserve could have a protective effect on cognitive decline, unfortunately proper evaluation was not possible due to lack of information beyond education and occupational status.

Our work highlights the current practice, aiming to diagnose these patients precisely and timely, to be able to start therapy early, and thus breaking the vicious circle of lesion formation and brain atrophy. Cognitive impairment is potentially very detrimental to MS patients’ level of function and quality of life.^33^ Precise clinical and MRI markers helping clinicians detecting patients at risk of cognitive decline, would be of great help, as it may aid treatment decisions.

## Conclusion

The growing awareness of cognitive difficulties in MS is essential for the patients and treating physicians, and identifying patients at risk of developing cognitive difficulties is key. The current study shows that both clinical and demographic charateristics is important in predicting ensuing cognitive difficulties, and that MRI parameters add to the explanatory model. Identifying patients at higher risk of developing cognitive difficulties could help clinicians initiate proper follow-up, and treatment decisions.

## Supplemental Material

sj-pdf-1-mso-10.1177_2055217321992394 - Supplemental material for Brain atrophy and clinical characteristics predicting SDMT performance in multiple sclerosis: A 10-year follow-up study Click here for additional data file. Supplemental material, sj-pdf-1-mso-10.1177_2055217321992394 for Brain atrophy and clinical characteristics predicting SDMT performance in multiple sclerosis: A 10-year follow-up study by Cecilie Jacobsen, Robert Zivadinov, Kjell-Morten Myhr, Turi O Dalaker, Ingvild Dalen, Ralph HB Benedict, Niels Bergsland and Elisabeth Farbu in Multiple Sclerosis Journal – Experimental, Translational and Clinical
