# Supplementary material for: Brain atrophy and clinical characteristics predicting SDMT performance in multiple sclerosis: A 10-year follow-up study
Source: Mult Scler J Exp Transl Clin. 2021 Feb 8;7(1):2055217321992394. doi: 10.1177/2055217321992394 (PMC7876764; doi:10.1177/2055217321992394)
Supplement: sj-pdf-1-mso-10.1177_2055217321992394 - Supplemental material for Brain atrophy and clinical characteristics predicting SDMT performance in multiple sclerosis: A 10-year follow-up study [file sj-pdf-1-mso-10.1177_2055217321992394.pdf]

Supplementary table 1. Prediction of clinically relevant reduction in SDMT during 5 years from baseline (31 cases, 35 non-cases)

	Univariable				Multivariable					
Baseline predictor	OR (95% CI)	p	C	R^2 (%)	OR (95% CI)	p	C	R^2 (%)	ΔC	ΔR^2
					Best model clin/dem					
Age	1.45 (0.85, 2.45)	0.17	0.60	3.9						
Sex (female)	0.63 (0.23, 1.77)	0.39	0.55	1.5						
Center (SUS)	6.07 (2.08, 17.66)	**0.001**	0.71	22.3	8.71 (2.55, 29.78)	**0.001**				
Education (high)	0.78 (0.28, 2.23)	0.65	0.53	0.4			0.81	36.9		
Log disease duration	1.62 (0.82, 3.19)	0.16	0.60	4.0	2.03 (0.91, 4.53)	0.083				
MS type progressive	1.41 (0.42, 4.76)	0.58	0.53	0.6						
EDSS	1.62 (1.06, 2.47)	**0.026**	0.64	10.9	1.72 (1.03, 2.88)	**0.037**				
SDMT	1.00 (0.97, 1.04)	0.90	0.51	0.0						
					When added to best model clin/dem					
Whole brain	0.989 (0.981, 0.996)	**0.003**	0.72	21.0	0.993 (0.984, 1.002)	0.11	0.83	40.9	0.02	4.0
Grey matter	0.987 (0.977, 0.998)	**0.015**	0.67	13.0	0.991 (0.978, 1.004)	0.17	0.82	39.9	0.01	3.0
White matter	0.980 (0.966, 0.994)	**0.006**	0.70	18.5	0.989 (0.972, 1.006)	0.20	0.82	39.4	0.01	2.5
Ventricular	1.00 (0.98, 1.03)	0.80	0.51	0.1	1.01 (0.98, 1.05)	0.40	0.81	38.0	0.00	1.1
Cortical	0.980 (0.967, 0.994)	**0.004**	0.70	19.6	0.988 (0.973, 1.004)	0.14	0.83	40.3	0.02	3.4
T2 LV	1.05 (1.00, 1.10)	**0.032**	0.67	10.0	1.07 (1.00, 1.14)	**0.043**	0.84	43.5	0.03	6.6
T1 LV	1.10 (1.01, 1.21)	**0.037**	0.70	10.4	1.11 (0.98, 1.26)	0.10	0.83	41.4	0.02	4.5
Subcortical	0.94 (0.86, 1.03)	0.19	0.59	3.6	0.99 (0.88, 1.12)	0.88	0.80	37.0	-0.01	0.1
Caudate	0.89 (0.53, 1.51)	0.67	0.56	0.4	1.35 (0.69, 2.64)	0.38	0.81	38.1	0.00	1.2
Putamen	0.87 (0.59, 1.27)	0.47	0.53	1.1	0.97 (0.58, 1.62)	0.90	0.80	37.0	-0.01	0.1
Thalamus	0.76 (0.57, 1.01)	0.059	0.65	7.5	0.88 (0.62, 1.26)	0.49	0.81	37.6	0.00	0.7
Pallidus	1.10 (0.38, 3.19)	0.86	0.50	0.1	1.46 (0.38, 5.62)	0.59	0.81	37.4	0.00	0.5
Hippocampus	0.77 (0.49, 1.21)	0.26	0.57	2.7	0.93 (0.54, 1.62)	0.81	0.80	37.0	-0.01	0.1
Amygdala	0.29 (0.09, 0.95)	**0.040**	0.63	9.2	0.75 (0.18, 3.07)	0.69	0.81	37.2	0.00	0.3

Legend: MS = Multiple Sclerosis (progressive=primary and secondary progressive combined); EDSS = Expanded Disability Status Scale; SDMT = Symbol Digit Modalities Test; LV = Lesion Volume; OR = Odds Ratio; CI = Confidence Interval.

Effect estimates from logistic regression analysis with reduction in SDMT greater than 4 points during 5 years (yes/no) as outcome, and baseline variables as predictors. An OR>1 means that a higher value of the predictor is associated with a greater odds of having a clinically significant decline in CPS. Predictive power of each model is estimated by the concordance index (C) and by Nagelkerke R^2, and the contribution of each predictor by the change in these statistics (ΔC and ΔR^2, respectively). Bold values denotes statistical significance at the $p< 0.05$ level.

Supplementary table 2. Prediction of clinically relevant reduction in SDMT during 10 years from baseline (13 cases, 37 non-cases)

	Univariable			
Predictor	OR (95% CI)	p	C	R^2(%)
Age	1.24 (0.61, 2.50)	0.55	0.55	1.1
Sex (female)	0.44 (0.11, 1.73)	0.24	0.58	3.9
Center (SUS)	1.69 (0.44, 6.39)	0.44	0.56	1.7
Education (high)	1.30 (0.35, 4.84)	0.69	0.53	0.5
Log disease duration	1.11 (0.48, 2.59)	0.80	0.62	0.2
MS type progressive	1.92 (0.39, 9.49)	0.42	0.55	1.8
EDSS	1.00 (0.62, 1.61)	1.00	0.51	0.0
SDMT	1.03 (0.97, 1.08)	0.33	0.59	2.8
Whole brain	0.997 (0.989, 1.005)	0.46	0.58	1.6
Grey matter	0.992 (0.981, 1.004)	0.19	0.60	5.3
White matter	1.003 (0.988, 1.017)	0.72	0.50	0.4
Ventricular	0.999 (0.970, 1.028)	0.92	0.51	0.0
Cortical	0.992 (0.978, 1.006)	0.24	0.60	4.3
T2 LV	1.05 (1.00, 1.10)	**0.044**	0.68	12.0
T1 LV	1.14 (1.01, 1.29)	**0.037**	0.72	12.9
Subcortical	0.95 (0.84, 1.07)	0.41	0.57	2.0
Caudate	0.97 (0.48, 1.95)	0.93	0.52	0.0
Putamen	0.83 (0.50, 1.39)	0.49	0.54	1.4
Thalamus	0.85 (0.59, 1.23)	0.39	0.59	2.2
Pallidus	0.61 (0.14, 2.71)	0.51	0.55	1.3
Hippocampus	0.75 (0.42, 1.36)	0.35	0.44	2.6
Amygdala	0.63 (0.15, 2.66)	0.53	0.51	1.2

Legend: MS = Multiple Sclerosis (progressive=primary and secondary progressive combined); EDSS = Expanded Disability Status Scale; SDMT = Symbol Digit Modalities Test; LV = Lesion Volume; OR = Odds Ratio; CI = Confidence Interval.

Effect estimates from logistic regression analysis with reduction in SDMT greater than 4 points during 5 years (yes/no) as outcome, and baseline variables as predictors. An OR>1 means that a higher value of the predictor is associated with a greater odds of having a clinically significant decline in CPS. Predictive power of each model is estimated by the concordance index (C) and by Nagelkerke R^2. Bold values denotes statistical significance at the $p< 0.05$ level.
